# Statistical properties of methods based on the *Q*‐statistic for constructing a confidence interval for the between‐study variance in meta‐analysis

**DOI:** 10.1002/jrsm.1336

**Published:** 2019-01-28

**Authors:** Robbie C.M van Aert, Marcel A.L.M. van Assen, Wolfgang Viechtbauer

**Affiliations:** ^1^ Department of Methodology and Statistics Tilburg University Tilburg the Netherlands; ^2^ Department of Sociology Utrecht University Utrecht the Netherlands; ^3^ Department of Psychiatry and Neuropsychology Maastricht University Maastricht the Netherlands

**Keywords:** confidence intervals, heterogeneity, meta‐analysis, random‐effects model

## Abstract

The effect sizes of studies included in a meta‐analysis do often not share a common true effect size due to differences in for instance the design of the studies. Estimates of this so‐called between‐study variance are usually imprecise. Hence, reporting a confidence interval together with a point estimate of the amount of between‐study variance facilitates interpretation of the meta‐analytic results. Two methods that are recommended to be used for creating such a confidence interval are the *Q*‐profile and generalized *Q*‐statistic method that both make use of the *Q*‐statistic. These methods are exact if the assumptions underlying the random‐effects model hold, but these assumptions are usually violated in practice such that confidence intervals of the methods are approximate rather than exact confidence intervals. We illustrate by means of two Monte‐Carlo simulation studies with odds ratio as effect size measure that coverage probabilities of both methods can be substantially below the nominal coverage rate in situations that are representative for meta‐analyses in practice. We also show that these too low coverage probabilities are caused by violations of the assumptions of the random‐effects model (ie, normal sampling distributions of the effect size measure and known sampling variances) and are especially prevalent if the sample sizes in the primary studies are small.

## 1 INTRODUCTION

Meta‐analysis refers to a set of statistical techniques for combining the estimates of similar studies providing commensurable evidence about some phenomenon of interest (eg, the effectiveness of a treatment, the size of a group difference, or the strength of the association between two variables). By combining the evidence, we aim to increase statistical power to find effects or relationships that individual studies may fail to detect. Moreover, by examining the variability in the estimates, we can draw more generalizable conclusions about the consistency of the effect or relationship over multiple studies and/or examine the degree to which effects or relationships vary and under what conditions.

If the included studies in a meta‐analysis share the same common true effect size, any differences between the studies' effect size estimates are in theory only caused by sampling variability. However, the true effect sizes can also vary and sampling variability alone can then not explain the differences in effect size estimates. The effect sizes are then said to be heterogeneous. Such between‐study variance may be due to systematic differences between the studies (eg, differences in the sample characteristics or differences in the length or dose of a treatment). If information on how the studies differ is available, it may be possible to account for the between‐study variance by incorporating this information in the model with a meta‐regression analysis.^1^

The *Q*‐test^2^ is commonly used to test the null hypothesis of no between‐study variance. A drawback of the *Q*‐test is that the test can have low statistical power if a small number of studies are included and can have very high power if a large number of studies are included even if the amount of variability in the true effects is negligible.^3^, ^5^ These undesirable statistical properties of the *Q*‐test call attention to the importance for estimating the amount of between‐study variance. The amount of between‐study variance as well as the average effect size of the set of studies can be estimated by means of a random‐effects model. Estimating the between‐study variance is equally important as estimating the average effect size because it indicates the amount of consistency among the effects.^6^ However, estimates of the between‐study variance are rather imprecise if the number of studies in a meta‐analysis is small.^7^, ^9^ Hence, reporting a confidence interval (CI) around the estimate is highly desirable and improves interpretability.^6^, ^10^, ^11^, ^12^

Numerous methods for constructing a CI around the estimate of the between‐study variance have been proposed, including the profile likelihood method,^13^ Wald‐type methods,^14^ bootstrapping,^15^, ^16^ a method by Sidik and Jonkman based on weighted least squares estimation,^17^ the *Q*‐profile method,^18^ two different methods that approximate the distribution of the test statistic of the *Q*‐test,^14^, ^19^, ^20^ and also Bayesian methods to estimate a corresponding credible interval.^21^ Since the method proposed by Biggerstaff and Jackson^19^ is a special case of the method described by Jackson,^20^ we will refer to this method as the generalized *Q*‐statistic method (GENQ method for short). A recent review of the aforementioned methods^22^ recommended to use the *Q*‐profile method if the between‐study variance is large and the GENQ method if the between‐study variance is small.

The *Q*‐profile and GENQ methods make use of the distribution of the test statistic of the *Q*‐test to compute a CI. If the assumptions underlying the random‐effects model hold, the null distribution of the *Q*‐statistic is *χ*^2^ with the number of studies minus one as the degrees of freedom.^2^ However, violations of these assumptions are likely to occur in practice. For instance, an assumption of the random‐effects model is that the sampling distribution of each study's effect size is normally distributed (see Jackson and White^23^ and the corresponding commentaries for a general discussion on normality assumptions in meta‐analysis). This assumption is violated in most meta‐analyses because the sampling distribution of most effect size measures is only asymptotically normal (ie, approximates a normal distribution as the primary study's sample size gets large).^4^, ^23^, ^24^, ^25^ Another assumption is that the sampling variances are known, whereas they are usually estimated and then simply assumed to be known.^14^, ^26^ These assumptions become more acceptable if the primary studies' sample sizes increase, because the sampling distributions are then better approximated by normal distributions and the primary studies' observed sampling variances are closer to the true sampling variances. Nevertheless, violations of the assumptions of the random‐effects model will result in a *Q*‐statistic that does not exactly follow a *χ*^2^ distribution under the null hypothesis. Hence, the *Q*‐profile and GENQ methods may not yield exact CIs (ie, coverage probability equal to 1 − α) if these assumptions do not hold.

The aim of our paper is to study the performance of the *Q*‐profile and GENQ methods under conditions that are representative for meta‐analyses in practice. We selected the log odds ratio as the effect size measure in our analyses, because it is often used in medical research. Note that the above discussed assumptions of normal sampling distributions and known sampling variances are violated if the log odds ratio is the effect size measure and that these violations can be substantial particularly if the primary studies' sample sizes are small. The statistical properties of the *Q*‐profile method have already been examined under conditions that are representative for meta‐analyses in practice where the assumptions of the random‐effects model are violated.^18^ However, statistical properties of the GENQ method have only been studied under conditions where all assumptions of the random‐effects model hold.^20^, ^27^ Our paper is therefore the first that compares the statistical properties of the *Q*‐profile and GENQ methods when the assumptions of the random‐effects model do not hold in combination with conditions that are representative for meta‐analysis in practice.

The paper continues by briefly outlining the random‐effects model and the *Q*‐test. Subsequently, the *Q*‐profile and GENQ methods are described and illustrated using a meta‐analysis on the relationship between handedness and eye‐dominance. Next, we describe the Monte‐Carlo simulation study that we use to examine the statistical properties of the two methods and present their results. The paper ends with a conclusion and discussion section with recommendations for when to use the *Q*‐profile and GENQ methods.

## 2 THE RANDOM‐EFFECTS MODEL AND *Q*‐TEST

Assume that *i =* 1, 2, … *k* independent effect sizes have been derived from a set of studies. Each study's observed effect size (*Y*_*i*_) is assumed to be an unbiased estimate of the study specific true effect size (*θ*_*i*_). However, *Y*_*i*_ is not equal to due to sampling error (*ε*_*i*_). This can be written as

$$Y_{i}=\theta_{i}+\varepsilon_{i}$$

where $\varepsilon_{i}\sim N\left(0,\sigma_{i}^{2}\right)$ with $\sigma_{i}^{2}$ denoting the true sampling variance in the *i*th study. All *ε*_*i*_ are assumed to be independent of each other and each $\sigma_{i}^{2}$ is estimated in practice and then assumed to be known. Hence, we will write ${\hat{\sigma }}_{i}^{2}$ to refer to the estimated sampling variances. Each *θ*_*i*_ consists of an average true effect (*μ*) and the random effect *u*_*i*_~*N*(0, *τ*^2^) that denotes the difference between *θ*_*i*_ and *μ*.^26^ Hence, the random‐effects model can be written as

$$Y_{i}=\mu +u_{i}+\varepsilon_{i}$$

where it is assumed that the *u*_*i*_ are independent of each other and *u*_*i*_ is independent of *ε*_*i*_. The random‐effects model reduces to the common‐ or equal‐effects model if *τ*^2^ = 0.

Several hypothesis tests for testing *H*_0_: *τ*^2^ = 0 have been proposed,^5^ of which the *Q*‐test is most often used.^25^ The *Q*‐statistic is computed with

(1) $$Q=\sum_{i=1}^{k}\frac{{\left(Y_{i}-\hat{\theta }\right)}^{2}}{{\hat{\sigma }}_{i}^{2}},$$

where $\hat{\theta }$ is given by

(2) $$\hat{\theta }=\frac{\sum_{i=1}^{k}w_{i}Y_{i}}{\sum_{i=1}^{k}w_{i}},$$

with $w_{i}=1/{\hat{\sigma }}_{i}^{2}$. Under the null hypothesis, *Q* follows a *χ*^2^distribution with *k* − 1 degrees of freedom if the primary studies' sample sizes are large.^2^ Hence, *H*_0_: *τ*^2^ = 0 is rejected when testing with α = 0.05 if *Q* is larger than $\chi_{k-1;0.95}^{2}$, where $\chi_{k-1;0.95}^{2}$ is the 95th percentile of a *χ*^2^ distribution with *k* − 1 degrees of freedom.

## 3 *Q*‐PROFILE METHOD

The *Q*‐profile method generalizes the *Q*‐statistic in Equation 1 to a random‐effects model by incorporating *τ*^2^, so that

(3) $$Q\left(\tau^{2}\right)=\sum_{i=1}^{k}\frac{{\left(Y_{i}-\hat{\mu }\right)}^{2}}{\tau^{2}+{\hat{\sigma }}_{i}^{2}},$$

with $\hat{\mu }$ given by Equation 2 with $w_{i}=1/\left(\tau^{2}+{\hat{\sigma }}_{i}^{2}\right)$. This generalized version of the *Q*‐statistic is a pivotal quantity that also follows a *χ*^2^ distribution with *k* − 1 degrees of freedom^18^ and is a function of *τ*^2^. Hence, a CI for *τ*^2^ can be obtained by means of test inversion.^28^ If $\chi_{k-1;0.025}^{2}$ and $\chi_{k-1;0.975}^{2}$ are the 2.5th and 97.5th percentiles of a *χ*^2^ distribution with *k* − 1 degrees of freedom, the 95% CI ( ${\hat{\tau }}_{\mathrm{LB}}^{2};{\hat{\tau }}_{\mathrm{UB}}^{2}$) is equal to the two values for *τ*^2^ where

$$\left(Q\left(\tau^{2}={\hat{\tau }}_{\mathrm{LB}}^{2}\right)=\chi_{k-1;0.975}^{2};Q\left(\tau^{2}={\hat{\tau }}_{\mathrm{UB}}^{2}\right)=\chi_{k-1;0.025}^{2}\right).$$

The method is called *Q*‐profile because different values for *τ*^2^ are entered in Equation 3 (ie, profiling) until the pivotal quantity in Equation 3 equals the critical values of the *χ*^2^ distribution. If $Q\left(\tau^{2}=0\right)<\chi_{k-1;0.975}^{2}$, the lower bound of the CI is in principle negative but outside of the parameter space and hence truncated to zero.^18^ If $Q\left(\tau^{2}=0\right)<\chi_{k-1;0.025}^{2}$, the estimate of the upper bound is also negative, and the CI is set equal to the null set. Under the assumptions of the random‐effects model (ie, unbiased observed effect size estimates, normal sampling distributions, known sampling variances, and uncorrelated sampling errors and random effects), the *Q*‐profile method yields exact CIs. Viechtbauer^29^ showed by means of a Monte‐Carlo simulation study with log odds ratios as effect size measure (which do not fulfill the model assumptions exactly) that the *Q*‐profile method still yields accurate coverage probabilities for the majority of the conditions included in the simulations. One exception was that undercoverage occurred when meta‐analyzing a large number of studies with small sample sizes.

## 4 GENQ METHOD

The GENQ method^19^, ^20^ constructs a CI for *τ*^2^ based on the exact distribution of the *Q*‐statistic under the assumptions of the random‐effects model. This method uses the generalized form of the *Q*‐statistic as described by DerSimonian and Kacker^30^ where the weights are no longer, $w_{i}=1/{\hat{\sigma }}_{i}^{2}$, but could be any set of positive constants denoted by *a*_*i*_. The exact distribution of the *Q*‐statistic (*Q*_*a*_) was derived by Biggerstaff and Jackson^19^ and Jackson.^20^ The distribution of *Q*_*a*_ is the weighted sum (weighted by *λ*_*i*_ ≥ 0 where *λ*_*i*_ are the eigenvalues of a matrix that is a function of *a*_*i*_, ${\hat{\sigma }}_{i}^{2}$, and *τ*^2^) of mutually independent *χ*^2^‐distributed random variables with one degree of freedom each, so that

(4) $$Q_{a}\overset{d}{=}\sum_{i=1}^{k}\lambda_{i}\chi_{i}^{2}\left(1\right).$$

Jackson^20^ proved that the cumulative distribution function of *Q*_*a*_ is a continuous and decreasing function in *τ*^2^. The cumulative distribution function of a positive linear combination of *χ*^2^‐distributed random variables can be obtained by Farebrother's algorithm.^31^ The lower and upper bound of the 95% CI ( ${\hat{\tau }}_{\mathrm{LB}}^{2};{\hat{\tau }}_{\mathrm{UB}}^{2}$) can then be obtained again by test inversion^28^; that is, given the observed value *q*_*a*_ of *Q*_*a*_, we find those two values of *τ*^2^ for which

$$\left(P\left(Q_{a}\geq q_{a};\tau^{2}={\hat{\tau }}_{\mathrm{LB}}^{2}\right)=0.025;P\left(Q_{a}\geq q_{a};\tau^{2}={\hat{\tau }}_{\mathrm{UB}}^{2}\right)=0.975\right).$$

The upper and lower bounds of the CI can also be negative. If the estimate of the lower bound is negative, it is recommended to truncate the estimate to zero. In case the lower and upper bounds are both negative, the CI is set equal to the null set. The GENQ method yields exact CIs if the assumptions underlying the random‐effects model (ie, unbiased observed effect size estimates, normal sampling distributions, known sampling variances, and uncorrelated standard errors and random effects) are fulfilled.

Different values for *a*_*i*_ can be selected for weighing the observed effect sizes. If $a_{i}=1/{\hat{\sigma }}_{i}^{2}$, the results of the methods by Biggerstaff and Jackson^19^ and Jackson^20^ are equivalent. Other suggestions for *a*_*i*_ are an unweighted analysis with *a*_*i*_ equal to a constant, $1/\left({\hat{\tau }}^{2}+{\hat{\sigma }}_{i}^{2}\right)$, and $1/{\left({\hat{\tau }}^{2}+{\hat{\sigma }}_{i}^{2}\right)}^{0.5}$.^20^, ^32^ Note that even when all model assumptions are fulfilled, the CIs are no longer exact if the last two weights are used, because the weights are then a function of a random variable (since *τ*^2^ has to be estimated).

## 5 EXAMPLE

We illustrate how the *Q*‐profile and GENQ methods can be used in practice by applying the methods to a meta‐analysis on the relationship between handedness and eye‐dominance by Bourassa et al.^33^ This meta‐analysis consists of 96 log odds ratios as effect size measure that were computed based on 2 × 2 frequency tables indicating the number of individuals that were left‐handed/left‐eyed, left‐handed/right‐eyed, right‐handed/right‐eyed, and right‐handed/left‐eyed. Data of the included 96 primary studies are reported in Table 1 of their paper. Before the log odds ratios were computed, we added 0.5 to each cell in the 2 × 2 frequency table of all primary studies in order to avoid division by zero when computing the log odds ratio and corresponding sampling variance and to decrease bias in the estimator of the log odds ratio.^34^ For the GENQ method, $a_{i}=1/{\hat{\sigma }}_{i}^{2}$ and $a_{i}=1/{\hat{\sigma }}_{i}$ were used as weights, because the method is exact if these weights are used and the model assumptions are fulfilled. R code for applying the *Q*‐profile and GENQ methods to these data is available at https://osf.io/s72r8/.

The *Q*‐statistic was equal to *Q*(95) = 561.06 (*p* < 0.0001), which implies that the null hypothesis of homogeneity was rejected. All 95% CIs of *Q*‐profile (0.268; 0.674), GENQ with $a_{i}=1/{\hat{\sigma }}_{i}^{2}$ (0.169; 0.633), and GENQ with $a_{i}=1/{\hat{\sigma }}_{i}$ (0.244; 0.622) did not include the value 0, suggesting that the true effect sizes were heterogeneous. However, considerable discrepancies among the methods are apparent for the lower bounds of the CIs.

## 6 MONTE‐CARLO SIMULATION STUDY 1

The *Q*‐profile and GENQ methods both yield exact CIs under the assumptions of the random‐effects model. However, these assumptions usually do not hold in practice but become more acceptable if the primary studies' sample sizes increase. Hence, the generalized *Q*‐statistics that are used for constructing the CIs with the *Q*‐profile and GENQ methods only approximate a *χ*^2^ distribution if the primary studies' sample sizes are large,^2^ and therefore, the CIs are really just approximations in practice instead of exact CIs. We will study the statistical properties of the CIs obtained with the *Q*‐profile and GENQ methods by means of two Monte‐Carlo simulation studies with the log odds ratio as effect size measure whose sampling distribution is only well approximated by a normal distribution for large sample sizes in the primary studies.

Data in both simulation studies were generated by first drawing the true log odds ratios, *θ*_*i*_ for *i* = 1, …, *k*, from *N*(*μ*, *τ*^2^), with *μ* denoting the mean of the distribution of the studies' true effect sizes and *τ*^2^ the variance of this distribution. Based on the sampled *θ*_*i*_, *k* 2 × 2 frequency tables were simulated by first generating the number of cases with the outcome of interest in the control group ( $x_{i}^{C}$). A value for $x_{i}^{C}$ was sampled from a binomial distribution with $n_{i}^{C}$ being the sample size of the control group and probability $\pi_{i}^{C}$ for the outcome of interest in the control group. A study's true log odds ratio (*θ*_*i*_) and $\pi_{i}^{C}$ were used for computing the probability of the outcome of interest in the experimental group with $\pi_{i}^{E}=\pi_{i}^{C}\exp\left(\theta_{i}\right)/\left[1-\pi_{i}^{C}+\pi_{i}^{C}\exp\left(\theta_{i}\right)\right]$. The number of cases with the outcome of interest in the experimental group, $x_{i}^{E}$, was sampled from a binomial ( $n_{i}^{E}$, $\pi_{i}^{E}$) distribution with $n_{i}^{E}$ being the total number of cases in the experimental group. Before computing the observed log odds ratio and corresponding sampling variance for each study, 0.5 was added to each cell of the frequency tables to decrease bias in the estimator of the log odds ratio.^34^ Furthermore, this adjustment allows calculation of the log odds ratio and its sampling variance in case of zero cells. Therefore, the observed log odds ratio was computed with

$$Y_{i}=\log\left[\frac{x_{i}^{E}+0.5}{n_{i}^{E}-x_{i}^{E}+0.5}/\frac{x_{i}^{C}+0.5}{n_{i}^{C}-x_{i}^{C}+0.5}\right]$$

and its observed sampling variance with

$${\hat{\sigma }}_{i}^{2}=\frac{1}{x_{i}^{E}+0.5}+\frac{1}{n_{i}^{E}-x_{i}^{E}+0.5}+\frac{1}{x_{i}^{C}+0.5}+\frac{1}{n_{i}^{C}-x_{i}^{C}+0.5}$$

The *Y*_*i*_ and ${\hat{\sigma }}_{i}^{2}$ values were used as input for the *Q*‐profile and GENQ methods. Two different weights were used for applying the GENQ method, $a_{i}=1/{\hat{\sigma }}_{i}^{2}$ and $a_{i}=1/{\hat{\sigma }}_{i}$ These two weights were selected because the GENQ method yields exact CIs for these two weights if the assumptions underlying the random‐effects model hold.

Values for the true effect size (*μ*) in this first simulation study were 0, 0.25, 0.5, 0.75, and 1. The amount of between‐study heterogeneity (*τ*) was varied between 0 and 0.5 with steps equal to 0.1, and three fixed values for $\pi_{i}^{C}$ were selected: 0.1, 0.3, and 0.5. For the condition with large heterogeneity (*τ* = 0.5) and *μ* = 0, the 95% prediction interval for *θ*_*i*_ ranges from −0.980 to 0.980, corresponding to odds ratios of 2.66 in favor of the control group to 2.66 in favor of the experimental group. The total number of observed effect sizes in a meta‐analysis (*k*) was 5, 10, 20, 40, and 160. Values for *k* are in line with previous Monte‐Carlo simulation studies^18^, ^20^ that examined the statistical properties of the *Q*‐profile and GENQ methods. We also included the condition *k* = 160 to examine the statistical properties of the methods for a very large number of studies. The sample size in the control and experimental group in each study was set equal to each other, but sample sizes were allowed to differ across the studies within a meta‐analysis. Sample sizes per group (30, 50, 100, 150, and 300) were replicated *k*/5 times in each meta‐analysis in order to hold the average sample size of the studies constant across conditions.

The outcome variables in our simulation study were the coverage probability (how often is *τ*^2^ in the CI of the *Q*‐profile and GENQ methods), the average width of the CI, the standard deviation (SD) of the width of the CI over all simulation runs, and the number of times the width of a particular method's CI was larger than the width of the other methods. We also stored for each simulation run the proportion of primary studies in the meta‐analysis that had one or two zeros in the 2 × 2 frequency table since coverage probabilities of the methods may especially deviate from the nominal coverage rate in these conditions. The simulations were programmed in R^35^ with 10 000 simulation runs per condition, and the Paule‐Mandel estimator for estimating *τ*^2^ was used when the *Q*‐profile method was applied since this estimator is one of the estimators that is nowadays recommended^12^, ^22^ and has the desirable property that its estimate is always inside the CI of the *Q*‐profile method. The *parallel* package^35^ was used to parallelize the computations, and the *metafor* package^36^ was used for applying the *Q*‐profile and GENQ methods. R code of this simulation study is available via https://osf.io/3x5rg/.

## 7 RESULTS MONTE‐CARLO SIMULATION STUDY 1

**Figure 1:**
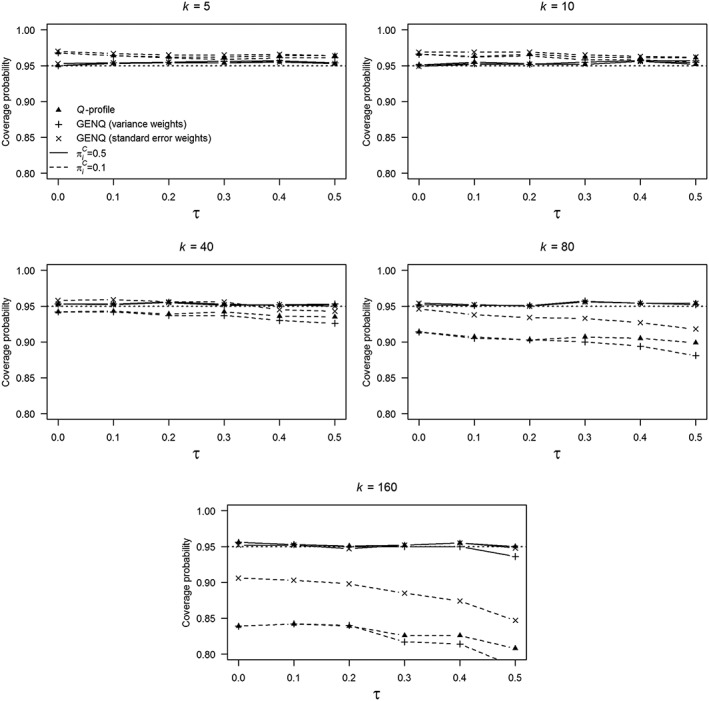
Coverage probabilities of the *Q*‐profile method, generalized *Q*‐statistic (GENQ) method with variance weights ( $a_{i}=1/{\hat{\sigma }}_{i}^{2}$), and GENQ method with standard error weights ( $a_{i}=1/{\hat{\sigma }}_{i}$). The probability of the outcome of interest in the control group is denoted by $\pi_{i}^{C}$, the number of primary studies in a meta‐analysis with *k*, and the amount of between‐study heterogeneity with *τ*

We only present the results for *μ* = 0, *k =* (5, 10, 40, 80, 160), and $\pi_{i}^{C}=\left(0.1,,,0.5\right)$, because these conditions are illustrative for the performance of the methods. Results were hardly affected by the selected values of *μ*, whereas results for $\pi_{i}^{C}=0.3$ were in between the two other conditions of $\pi_{i}^{C}$. Results of all other conditions are available via https://osf.io/qjv5x/. We will refer to the two different weights used for the GENQ method as *variance weights* for $a_{i}=1/{\hat{\sigma }}_{i}^{2}$ and *standard error weights* for $a_{i}=1/{\hat{\sigma }}_{i}$. Figure 1 shows the coverage probabilities of these two methods and the *Q*‐profile method as a function of true heterogeneity *τ*. The solid lines refer to coverage probabilities for $\pi_{i}^{C}=0.5$ and the dashed lines to the coverage probabilities for $\pi_{i}^{C}=0.1$. Coverage probabilities of the *Q*‐profile method are indicated with triangles, the GENQ method with variance weights with plus signs, and the GENQ method with standard error weights with crosses. Note that we concluded that *τ* was not included in the CI if a CI was equal to the null set. Hence, coverage probabilities of the methods equal to 0.95 indicate nominal coverage for all conditions.

For all values of *k*, coverage probabilities of the *Q*‐profile and GENQ methods for $\pi_{i}^{C}=0.5$ were equal or close to 0.95. However, coverage of the methods for $\pi_{i}^{C}=0.1$ and *k =* 5 or 10 was slightly too large especially for τ = 0. Since coverage probabilities decreased when *k* was increased, coverage probabilities were reasonably close to the nominal coverage rate for *k =* 40 and $\pi_{i}^{C}=0.1$, but undercoverage and severe undercoverage were observed for *k =* 80 and 160 if $\pi_{i}^{C}=0.1$, respectively.

The lowest coverage probability for all methods was obtained in the condition *k =* 160, $\pi_{i}^{C}=0.1$, and *τ* = 0.5; for *Q*‐profile 0.808, GENQ with variance weights 0.782, and GENQ with standard error weights 0.847. For this condition, the undercoverage was fully explained by the upper bounds of the CIs being smaller than *τ* suggesting that the generalized *Q*‐statistic computed by replacing *τ*^2^ in Equation 3 with ${\hat{\tau }}^{2}$ was too low. This also explains why the undercoverage for $\pi_{i}^{C}=0.1$ and *k* = 160 was least severe for the GENQ method with standard error weights. Large (both positive and negative) effect sizes go together with unequally distributed cases in the 2 × 2 frequency table and thus large sampling variances. Equation 3 shows that effect sizes that deviate substantially from $\hat{\mu }$ have only a minimal contribution to the generalized *Q*‐statistic because of their large sampling variance. If standard error weights are used instead of variance weights, more extreme effect sizes contribute more to the generalized *Q*‐statistic resulting in larger values for this statistic. Hence, undercoverage was less severe for the GENQ method with standard error weights than with variance weights.

Primary studies' 2 × 2 frequency tables containing one zero cell did not frequently occur for $\pi_{i}^{C}=0.5$ and $\pi_{i}^{C}$ = 0.3 (the proportion of simulation runs containing at least one primary study in a meta‐analysis with a zero cell was at most 0.077 across conditions). We examined whether the presence of primary studies with zero cells had an effect on the coverage probability by computing the methods' coverage on the subset of simulation runs containing at least one primary study in the meta‐analysis with a zero cell (*subset zero*) and on the subset of simulation runs without primary studies with a zero cell in the meta‐analysis (*subset nonzero*). Table S1 shows the coverage probabilities based on these subsets (columns *Coverage zero* and *Coverage nonzero*) together with the proportion of simulation runs in subset zero (column *Zero*) for the condition $\pi_{i}^{C}=0.1$. The proportion of simulation runs in subset zero increased as a function of *k* and *τ* and approached 1 for *k* = 160 and *τ* = 0.5. The coverage of the *Q*‐profile and GENQ methods with variance weights was closer to the nominal coverage rate for subset nonzero compared with subset zero for *k* < 40. This was the other way around for *k* > 40 where coverage of all methods for subset nonzero was smaller than coverage for subset zero implying severe undercoverage. The proportion of simulation runs containing at least one primary study in a meta‐analysis with two zeros was at most 0.09, and therefore, we did not separately study the coverage probabilities of the methods in these situations.

We also examined whether bias in the estimator of *μ* was related to deviations from the nominal coverage rate of the three methods. We computed the product‐moment correlation between the bias and a method's coverage rate across all conditions. These product‐moment correlations were −0.18, −0.227, and −0.258 for the *Q*‐profile, GENQ method with variance weights, and GENQ with standard error weights, respectively. These results imply that there was a small to medium negative relationship between bias in the estimator of *μ* and the methods' coverage rate, meaning that lower coverage was associated to overestimation of *μ*.

Table 1 presents the average and the SD of the width of a method's CI over all simulation runs. Bold values indicate the method with the smallest average width of the CI within a particular condition. As expected, the average width of the CIs decreased as a function of *k*. Coverage probabilities of the methods were in general close to the nominal coverage rate for $\pi_{i}^{C}=0.5$, so the method with the smallest CI is preferred in this condition. The CI of the GENQ method with variance weights was the smallest for the majority of the conditions. With the exception of one condition (ie, *k* = 20 and *τ* = 0.5), the average width of the CI for $\pi_{i}^{C}=0.5$ of the *Q*‐profile method was larger than of the GENQ methods. However, the difference between the method with the smallest and largest average width of a CI was at most 0.1 for *τ* ≤ 0.1 and at most 0.05 for *τ* > 0.1.

**Table 1:** Average and standard deviation (in parentheses) of the confidence interval width of the *Q*‐profile method, GENQ method with variance weights ( $a_{i}=1/{\hat{\sigma }}_{i}^{2}$), and GENQ method with standard error weights (SE; $a_{i}=1/{\hat{\sigma }}_{i}$)

		$\pi_{i}^{C}=0.5$					
		*τ* = 0	*τ* = 0.1	*τ* = 0.2	*τ* = 0.3	*τ* = 0.4	*τ* = 0.5
*k* = 5	*Q*‐profile	0.830 (0.392)	0.870 (0.398)	0.980 (0.405)	1.121 (0.433)	1.280 (0.454)	1.449 (0.501)
	GENQ (variance)	**0.747 (0.308)**	**0.797 (0.321)**	**0.934 (0.355)**	1.103 (0.402)	1.290 (0.450)	1.478 (0.512)
	GENQ (SE)	0.775 (0.332)	0.817 (0.340)	0.942 (0.359)	**1.097 (0.393)**	**1.267 (0.416)**	**1.438 (0.460)**
*k* = 10	*Q*‐profile	0.461 (0.188)	0.490 (0.186)	0.564 (0.175)	0.641 (0.161)	0.710 (0.155)	0.786 (0.166)
	GENQ (variance)	**0.404 (0.141)**	**0.440 (0.144)**	**0.531 (0.142)**	**0.623 (0.134)**	**0.709 (0.139)**	0.802 (0.167)
	GENQ (SE)	0.435 (0.156)	0.465 (0.156)	0.546 (0.152)	0.636 (0.136)	**0.709 (0.120)**	**0.783 (0.127)**
*k* = 40	*Q*‐profile	0.221 (0.075)	0.251 (0.068)	0.285 (0.040)	0.282 (0.029)	0.299 (0.030)	0.327 (0.034)
	GENQ (variance)	**0.199 (0.059)**	**0.230 (0.054)**	**0.267 (0.027)**	**0.269 (0.015)**	**0.294 (0.026)**	0.332 (0.035)
	GENQ (SE)	0.227 (0.069)	0.255 (0.064)	0.3 (0.041)	0.295 (0.023)	0.295 (0.015)	**0.318 (0.024)**
*k* = 80	*Q*‐profile	0.167 (0.052)	0.198 (0.043)	0.201 (0.022)	0.191 (0.014)	0.205 (0.015)	0.225 (0.017)
	GENQ (variance)	**0.154 (0.043)**	**0.185 (0.036)**	**0.187 (0.019)**	**0.181 (0.007)**	0.201 (0.013)	0.228 (0.017)
	GENQ (SE)	0.180 (0.051)	0.207 (0.045)	0.226 (0.029)	0.196 (0.011)	**0.200 (0.007)**	**0.218 (0.012)**
*k* = 160	*Q*‐profile	0.130 (0.039)	0.161 (0.027)	0.137 (0.011)	0.133 (0.007)	0.143 (0.007)	0.157 (0.008)
	GENQ (variance)	**0.122 (0.034)**	**0.153 (0.024)**	**0.126 (0.010)**	**0.125 (0.003)**	0.140 (0.006)	0.159 (0.009)
	GENQ (SE)	0.145 (0.040)	0.174 (0.032)	0.158 (0.024)	0.134 (0.002)	**0.139 (0.004)**	**0.152 (0.006)**

		$\pi_{i}^{C}=0.1$					
		*τ* = 0	*τ* = 0.1	*τ* = 0.2	*τ* = 0.3	*τ* = 0.4	*τ* = 0.5
*k* = 5	*Q*‐profile	1.399 (0.712)	1.412 (0.714)	1.488 (0.728)	1.587 (0.751)	1.732 (0.759)	1.889 (0.793)
	GENQ (variance)	**1.201 (0.500)**	**1.233 (0.518)**	**1.315 (0.537)**	**1.449 (0.589)**	**1.614 (0.627)**	**1.791 (0.682)**
	GENQ (SE)	1.276 (0.559)	1.297 (0.569)	1.372 (0.581)	1.486 (0.618)	1.639 (0.641)	1.807 (0.682)
*k* = 10	*Q*‐profile	0.734 (0.311)	0.760 (0.314)	0.814 (0.317)	0.899 (0.316)	0.981 (0.306)	1.067 (0.306)
	GENQ (variance)	**0.626 (0.223)**	**0.655 (0.23)**	**0.718 (0.239)**	**0.814 (0.247)**	**0.912 (0.243)**	**1.005 (0.241)**
	GENQ (SE)	0.696 (0.256)	0.722 (0.259)	0.774 (0.264)	0.862 (0.272)	0.955 (0.266)	1.051 (0.262)
*k* = 40	*Q*‐profile	0.317 (0.113)	0.337 (0.114)	0.395 (0.11)	0.455 (0.087)	0.474 (0.065)	0.477 (0.055)
	GENQ (variance)	**0.289 (0.094)**	**0.310 (0.096)**	**0.37 (0.093)**	**0.429 (0.069)**	**0.447 (0.042)**	**0.448 (0.028)**
	GENQ (SE)	0.348 (0.111)	0.365 (0.111)	0.417 (0.109)	0.485 (0.094)	0.526 (0.071)	0.525 (0.053)
*k* = 80	*Q*‐profile	0.223 (0.082)	0.246 (0.082)	0.307 (0.075)	0.347 (0.047)	0.330 (0.035)	0.321 (0.026)
	GENQ (variance)	**0.210 (0.073)**	**0.233 (0.072)**	**0.295 (0.066)**	**0.332 (0.041)**	**0.309 (0.028)**	**0.299 (0.013)**
	GENQ (SE)	0.263 (0.085)	0.282 (0.084)	0.336 (0.08)	0.396 (0.059)	0.395 (0.049)	0.354 (0.036)
*k* = 160	*Q*‐profile	0.160 (0.060)	0.183 (0.061)	0.249 (0.050)	0.256 (0.032)	0.223 (0.016)	0.221 (0.012)
	GENQ (variance)	**0.154 (0.055)**	**0.178 (0.056)**	**0.243 (0.046)**	**0.245 (0.034)**	**0.207 (0.012)**	**0.205 (0.005)**
	GENQ (SE)	0.199 (0.067)	0.219 (0.067)	0.279 (0.059)	0.322 (0.042)	0.271 (0.039)	0.237 (0.012)

Abbreviation: GENQ, generalized *Q*‐statistic.

The probability of having the outcome of interest in the control group is denoted by $\pi_{i}^{C}$, the number of primary studies in a meta‐analysis with *k*, and the amount of between‐study heterogeneity with *τ*.

The SDs of the width of the methods' CIs over all simulation runs were similar for $\pi_{i}^{C}=0.5$ and *k* < 160; the method with the highest SD never had a SD that was more than twice as large as the SD of the method with the smallest SD. The width of the CIs obtained with the GENQ method with variance weights was in at most 93.8% and 100% of the conditions smaller than that of the *Q*‐profile and GENQ methods with standard error weights, whereas the width of the CIs obtained with the *Q*‐profile method was in at most 59.3% and 98.3% of the conditions smaller than that of the GENQ method with variance and standard error weights, respectively. To summarize the results for $\pi_{i}^{C}=0.5$, the GENQ method with variance weights outperformed the other two methods for *τ* ≤ 0.3 in the majority of the conditions, and the GENQ method with standard error weights had the best statistical properties if *τ* > 0.3 in the majority of the conditions.

Results for $\pi_{i}^{C}=0.1$ are also presented in Table 1 but can hardly be interpreted. Coverage probabilities for these conditions often substantially deviated from the nominal coverage rate. Hence, drawing conclusions based on the width of a CI is not informative. Noteworthy though is that the GENQ method with variance weights always yielded smaller CIs than the *Q*‐profile and GENQ methods with standard error weights. Based on the results for $\pi_{i}^{C}=0.1$, we conclude that the GENQ method with standard error weights performs best, because its undercoverage is considerably less than that of the other two methods.

We created heat maps to gain further insight into whether there is a specific set of conditions for *k,* *τ*, $\pi_{i}^{C}$, $n_{i}^{E}$, and $n_{i}^{C}$ for which the coverage probability substantially diverges from the nominal coverage rate. For these conditions, researchers should be reluctant in applying these methods and interpreting their results. The heat maps show the coverage probabilities for different values of *k* (5, 10, 20, 40, 80, and 160) and $\pi_{i}^{C}$ ranging from 0.01 to 0.5 at a fixed sample size of 30 in both groups (ie, $n_{i}^{E}=n_{i}^{C}=30$). We also created heat maps in the same conditions but with $n_{i}^{E}$ and $n_{i}^{C}$ both being equal to either 15, 30, 80, 160, 320, or 800 while fixing *k* to 20. The heat maps were created for each of the three methods for *τ* = 0 and *τ* = 0.5. The procedure for creating the heat maps as well as the heat maps themselves is available via https://osf.io/e35qc/.

The heat maps confirmed the results presented in Figure 1 that *τ* only had a small effect on the coverage probabilities of the methods. Coverage probabilities decreased if $\pi_{i}^{C}$ decreased, and if undercoverage was present for a combination of $\pi_{i}^{C}$ and sample size, then this undercoverage became more severe as *k* increased. Furthermore, coverage probabilities also decreased if the sample size decreased, because the sampling variances were then less accurately estimated. The maximum coverage probability was equal to 0.97, so no severe overcoverage was observed. Specifically, coverage probabilities of all three methods were acceptable (ie, > 0.9) at a fixed sample size of 30 in both groups when *k =* 5 or 10 and $\pi_{i}^{C}\geq 0.05$, *k =* 20 and $\pi_{i}^{C}\geq 0.1$, *k =* 40 or 80 and $\pi_{i}^{C}\geq 0.2$, and *k =* 160 and $\pi_{i}^{C}\geq 0.35$. If *k* was fixed to 20, coverage probabilities were acceptable for $n_{i}^{E}$ = $n_{i}^{C}$ = 15 and $\pi_{i}^{C}\geq 0.2$, $n_{i}^{E}$ = $n_{i}^{C}$ = 30 and $\pi_{i}^{C}\geq 0.1$, and $n_{i}^{E}$ = $n_{i}^{C}$ = 80 and $\pi_{i}^{C}\geq 0.05$. The finding that coverage probabilities deviate from the nominal coverage rate for low values of $\pi_{i}^{C}$ and not for $\pi_{i}^{C}$ close to 0.5 hints at a systematic bias that is caused by violated assumptions of the random‐effects model in case of rare events in the primary studies. This bias will be examined in Monte‐Carlo simulation study 2.

## 8 MONTE‐CARLO SIMULATION STUDY 2

Monte‐Carlo simulation study 1 showed that coverage probabilities of both the *Q*‐profile and GENQ methods can substantially deviate from the nominal coverage rate. The goal of Monte‐Carlo simulation study 2 was to examine the cause of under‐ and overcoverage by the methods that was apparent for $\pi_{i}^{C}=0.1$ but not for $\pi_{i}^{C}=0.5$.

The *Q*‐profile and GENQ methods with the specified weights are exact if the assumptions underlying the random‐effects model hold, so deviations from the nominal coverage rate in Monte‐Carlo simulation study 1 were caused by violations of assumptions of the random‐effects model. One of the assumptions that is violated is that the primary studies' sampling variances are not known but estimated, which particularly affects the methods' coverage if the studies' sample sizes are small. Hence, we set out to compare the methods' coverage rates and the distribution of the generalized *Q*‐statistic used by the *Q*‐profile method when the sampling variances are estimated as in simulation study 1 (denoted by ${\hat{\sigma }}^{2}$) and when the true variances are used.

In order to compute the true variances, we first created all possible 2 × 2 frequency tables based on $n_{i}^{C}$ and $n_{i}^{E}$ given a particular value for $\pi_{i}^{C}$ and $\pi_{i}^{E}$. For example, this yields 31 × 31 = 961 possible frequency tables if the sample size in both groups was equal to 30. A selection of these 961 frequency tables is presented in Table 2 (first four columns). The probability of observing a particular frequency table (fifth column) was computed by multiplying *B*(*x*^*E*^; *n*^*E*^, *π*^*E*^) with *B*(*x*^*C*^; *n*^*C*^, *π*^*C*^), where *B* refers to the probability mass function of the binomial distribution. Log odds ratios (last column) were computed for each frequency table after adding 0.5 to each cell to reduce bias in the estimator of the log odds ratios^34^ and to make computation of the log odds ratio possible in all tables, even those with zero cells. We used the probability of observing a frequency table and the log odds ratio for each frequency table for computing the expected value of the log odds ratio (*E*[*Y*]) and the true sampling variance ( $\sigma_{T}^{2}=E\left[Y^{2}\right]-E\left[Y\right]$). We expect that the methods' coverage probabilities computed with $\sigma_{T}^{2}$ will be closer than the nominal coverage rate than with ${\hat{\sigma }}^{2}$, because instead of using estimated sampling variances, the true variances are used. Differences between ${\hat{\sigma }}^{2}$ and $\sigma_{T}^{2}$ are especially prevalent if one of the cells in the observed frequency table is equal to 0.

**Table 2:** Selection of all possible 2 × 2 frequency tables, probabilities of observing a table *B*(*x*^*E*^; *n*^*E*^, *π*^*E*^) × *B*(*x*^*C*^; *n*^*C*^, *π*^*C*^) with *B* denoting the probability mass function of the binomial distribution, and log odds ratios (*Y*) if the sample size in the experimental and control group equals 30

*x*^*E*^	*n*^*E*^ − *x*^*E*^	*x*^*C*^	*n*^*C*^ − *x*^*C*^	*B*(*x*^*E*^; *n*^*E*^, *π*^*E*^) × *B*(*x*^*C*^; *n*^*C*^, *π*^*C*^)	*Y*
0	30	0	30	*B*(0; 30, *π*^*E*^) × *B*(0; 30, *π*^*C*^)	0
1	29	0	30	*B*(1; 30, *π*^*E*^) × *B*(0; 30, *π*^*C*^)	1.132
2	28	0	30	*B*(2; 30, *π*^*E*^) × *B*(0; 30, *π*^*C*^)	1.677
3	27	0	30	*B*(3; 30, *π*^*E*^) × *B*(0; 30, *π*^*C*^)	2.049
4	26	0	30	*B*(4; 30, *π*^*E*^) × *B*(0; 30, *π*^*C*^)	2.338
⁞	⁞	⁞	⁞	⁞	⁞
0	30	1	29	*B*(0; 30, *π*^*E*^) × *B*(1; 30, *π*^*C*^)	−1.132
⁞	⁞	⁞	⁞	⁞	⁞
30	0	30	0	*B*(30; 30, *π*^*E*^) × *B*(30; 30, *π*^*C*^)	0

Cell frequencies are denoted by *x*^*E*^, *n*^*E*^ − *x*^*E*^, *x*^*C*^, and *n*^*C*^ − *x*^*C*^.

The computation time of $\sigma_{T}^{2}$ was large, and therefore we could not include the same conditions as in Monte‐Carlo simulation study 1. One value for the true effect size was selected (*μ* = 0), $\pi_{i}^{C}$ was 0.1 or 0.5, and *k* was set equal to 5, 40, or 160. The sample size of all studies was set equal to 30, because the methods' coverage probabilities were expected to deviate the most from the nominal coverage rate in this condition with the smallest study sample sizes. The amount of between‐study heterogeneity (*τ*) was, as in Monte‐Carlo simulation study 1, varied from 0 to 0.5 in steps of 0.1. This simulation study was also programmed in R^35^ and the packages *parallel* 35 and *metafor* 36 were used. A total number of 3000 simulation runs per condition were used. R code of simulation study 2 is available via https://osf.io/xba4y/.

## 9 RESULTS MONTE‐CARLO SIMULATION STUDY 2

**Figure 2:**
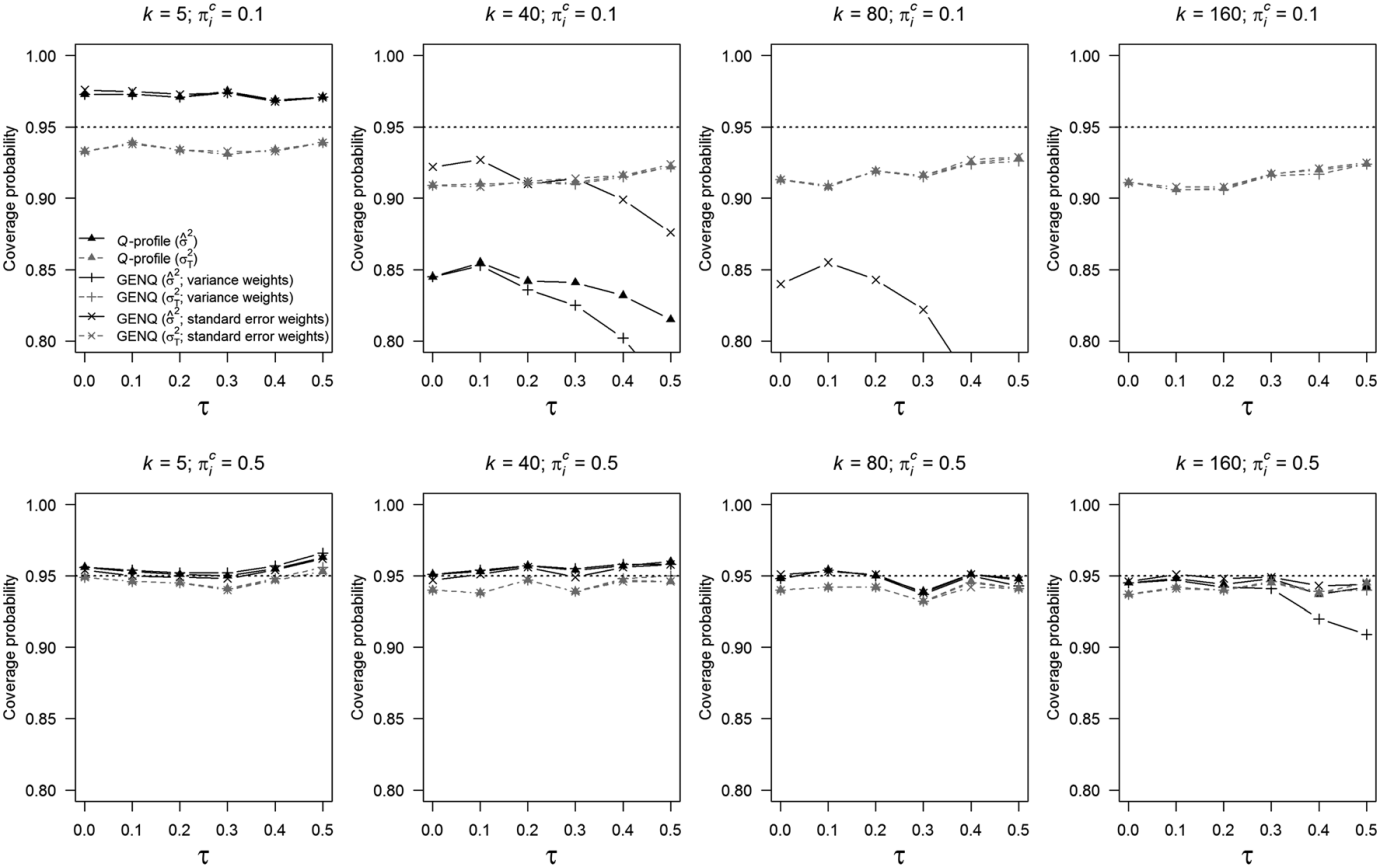
Coverage probabilities of the *Q*‐profile method, generalized *Q*‐statistic (GENQ) method with variance weights, and GENQ method with standard error weights. The probability of the outcome of interest in the control group is denoted by $\pi_{i}^{C}$, the number of primary studies in a meta‐analysis with *k*, and the amount of between‐study heterogeneity with *τ*. The estimator of the sampling variance ( ${\hat{\sigma }}^{2}$) is indicated with solid black lines and the true sampling variance ( $\sigma_{T}^{2}$) with dashed gray lines. Note that the methods' coverage probabilities in the condition with estimated sampling variances are not shown in the upper right panel for *k* = 160 and $\pi_{i}^{C}=0.1$, since all these coverage probabilities were too low (<0.8)

Figure 2 shows the coverage probabilities of the *Q*‐profile and GENQ methods when using estimator ${\hat{\sigma }}^{2}$ and when using the true variances $\sigma_{T}^{2}$. Similar to Figure 1, triangles refer to the *Q*‐profile method, plus signs to the GENQ method with variance weights, and crosses to the GENQ method with standard error weights. The estimator of the sampling variance that was also used in Monte‐Carlo simulation study 1 ( ${\hat{\sigma }}^{2}$) is indicated with solid black lines, while $\sigma_{T}^{2}$ is indicated with dashed gray lines. Note that the results of both simulation studies cannot directly be compared because a sample size of 30 for each primary study was used in Monte‐Carlo simulation study 2 instead of different sample sizes as in simulation study 1. All results of this simulation study are also available at https://osf.io/kzsv3/.

In general, coverage probabilities were closer to the nominal coverage rate if $\pi_{i}^{C}=0.1$ and $\sigma_{T}^{2}$ was used. This can be seen in the top left panel of Figure 2 (*k =* 5; $\pi_{i}^{C}=0.1$) where coverage probabilities were closer to the nominal coverage rate (although slightly too low) when $\sigma_{T}^{2}$ was used instead of ${\hat{\sigma }}^{2}$. If $\pi_{i}^{C}=0.5$ (second row of panels in Figure 2), no severe undercoverage was for the three methods when using ${\hat{\sigma }}^{2}$ or $\sigma_{T}^{2}$ since all coverage probabilities were larger than 0.9. Monte‐Carlo simulation study 1 showed that coverage probabilities most notably diverged from the nominal coverage rate when *k* = 160 and $\pi_{i}^{C}=0.1$. This is also apparent here; coverage probabilities based on ${\hat{\sigma }}^{2}$ are below 0.8 for each value of *τ* and therefore not visible in the figure. Coverage probabilities of the *Q*‐profile and GENQ methods with variance weights were not above 0.265, and coverage probabilities of the GENQ method with standard error weights were not above 0.657. However, although coverage probabilities substantially improved when using $\sigma_{T}^{2}$ (eg, for *τ* = 0.5 *Q*‐profile: 0.220 vs 0.924, GENQ with variance weights: 0.115 vs 0.924, and GENQ with standard error weights: 0.471 vs 0.925), coverage probabilities still deviated from the nominal coverage rate.

We also examined whether coverage probability of the methods when using ${\hat{\sigma }}^{2}$ was related to the presence of zero cells in the primary studies included in the meta‐analysis. Table S2 shows the proportion of simulation runs in subset zero (*Zero*), its coverage probabilities (*Coverage zero*), and these probabilities for subset nonzero (*Coverage nonzero*). The proportion of simulation runs in subset zero was large because the sample size per group was small and fixed to 30 for all studies in the meta‐analysis. The results show that the proportion of simulation runs in subset zero was large for *k* > 5 (at least 0.967). Coverage probabilities of all methods were closer to the nominal coverage rate in subset nonzero for *k* = 5, but for *k* = 40, the methods' undercoverage was less severe for subset zero. Table S3 shows the same results as in Table S2 but then for meta‐analyses containing at least one primary study with two zero cells (subset two zeros). These results show that the methods' coverage for subset two zeros was generally closer to the nominal coverage rate than for meta‐analyses without at least one primary study containing two zero cells. To conclude, using true sampling variances rather than estimated sampling variances considerably improved the coverage probability of the *Q*‐profile and GENQ methods but did not always provide nominal CIs. It follows that these deficiencies must be caused by two other assumptions of the random‐effects model that were violated in our simulation study; normal sampling distributions of the effect sizes and uncorrelated random effects and sampling errors.

To increase our understanding of how violating the assumption of known sampling variances as well as violations of other assumptions underlying the random‐effects model affect the generalized *Q*‐statistic, we computed the generalized *Q*‐statistic as described in Equation 3 based on ${\hat{\sigma }}^{2}$ and $\sigma_{T}^{2}$ and examined how well its probability density function (pdf) was approximated by a *χ*^2^ distribution with *k* − 1 degrees of freedom. Since the generalized *Q*‐statistic follows a *χ*^2^ distribution if the assumptions of the random‐effects model hold and these assumptions become less objectionable for larger sample sizes in the primary studies, we also computed the pdf with ${\hat{\sigma }}^{2}$ if there were 10 times more cases in the control and experimental group (300 instead of 30). Three different conditions were selected, representing coverage probabilities of the methods equal to the nominal coverage rate (*k* = 5, $\pi_{i}^{C}=0.5$, *τ* = 0), overcoverage (*k* = 5, $p_{i}^{C}$ = 0.1, τ = 0), and undercoverage (*k* = 160, $\pi_{i}^{C}=0.1$, *τ* = 0.5). Pdfs were created based on 5000 generated generalized *Q*‐statistics and R code for creating these pdfs is available via https://osf.io/bdhn8/. We focus in this section on the approximation of the generalized *Q*‐statistic by the *χ*^2^ distribution in the *Q*‐profile method, because *Q*_*a*_ as used by the GENQ methods depends on the weights *λ*_*i*_ (see Equation 4) and therefore does not follow a single reference distribution. However, because coverage probabilities of the GENQ method with variance weights and the *Q*‐profile method were comparable, we expect similar deviations of the weighted *χ*^2^ distribution for the GENQ method as the deviations we find for *Q*‐profile method.

**Figure 3:**
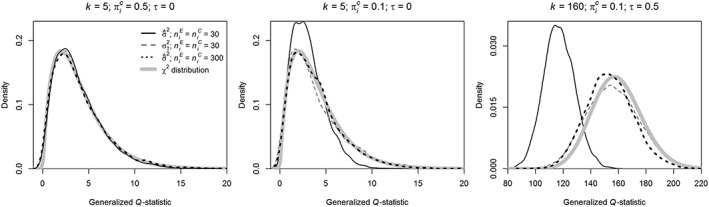
Probability density functions (pdfs) of the generalized *Q*‐statistic (Equation 3) for *k* = 5, $\pi_{i}^{C}=0.5$, and *τ* = 0 (coverage probability equal to nominal coverage rate); *k* = 5, $\pi_{i}^{C}=0.1$, and *τ* = 0 (overcoverage); and *k* = 160, $\pi_{i}^{C}=0.1$, and *τ* = 0.5 (undercoverage). Pdfs based on a sample size of 30 in the experimental and control group were obtained with three different estimators for the sampling variance: ${\hat{\sigma }}^{2}$ (solid black line) and $\sigma_{T}^{2}$ (dashed gray line). The pdf based on a sample size of 300 in both groups with estimator ${\hat{\sigma }}^{2}$ is presented with the dotted black line. The pdf of the *χ*^2^ statistic is denoted by the bold gray line

Figure 3 shows the pdfs of the generalized *Q*‐statistic when the coverage probability was close to the nominal coverage rate (left panel; *k* = 5, $\pi_{i}^{C}=0.5$, *τ* = 0), when coverage was too large (middle panel; *k* = 5, $\pi_{i}^{C}=0.1$, *τ* = 0), and when coverage was too low (right panel; *k* = 160, $\pi_{i}^{C}=0.1$, *τ* = 0.5). The pdf of the generalized *Q*‐statistic when the sampling variance is computed with ${\hat{\sigma }}^{2}$ is illustrated with a solid black line and $\sigma_{T}^{2}$ with a dashed gray line. The bold gray line corresponds to a *χ*^2^ distribution with *k* − 1 degrees of freedom, which in theory should be the distribution that is approximated by the other pdfs. Starting with the left panel (close to accurate coverage; *k* = 5, $\pi_{i}^{C}=0.5$, *τ* = 0), the mean of the generalized *Q*‐statistics was indeed close to the mean (4) of the *χ*^2^ distribution (3.86 for ${\hat{\sigma }}^{2}$, 3.96 for $\sigma_{T}^{2}$). However, the variance (4 × 2 = 8) was somewhat different for ${\hat{\sigma }}^{2}$ (6.73), but not for $\sigma_{T}^{2}$ (7.69). As expected, the pdf was closely approximated by the *χ*^2^ distribution if the primary studies' sample size was equal to 300 and ${\hat{\sigma }}^{2}$ was used to estimate the sampling variance (mean 4.01 and variance 8.20). These results suggest that the sampling variance was accurately estimated with ${\hat{\sigma }}^{2}$ for *k* = 5, $\pi_{i}^{C}=0.5$, and *τ* = 0, and that the sample size of 30 was sufficiently large for this condition to approximate the pdf of the generalized *Q*‐statistic with a *χ*^2^ distribution.

The pdfs of the generalized *Q*‐statistic for the condition with overcoverage (*k* = 5, $\pi_{i}^{C}=0.1$, *τ* = 0) are presented in the middle panel of Figure 3. The pdf of the generalized *Q*‐statistic based on $\sigma_{T}^{2}$ was closer to the *χ*^2^ distribution than based on ${\hat{\sigma }}^{2}$. Especially the variance of the generalized *Q*‐statistic based on ${\hat{\sigma }}^{2}$ was too low (mean 3.07 < 4, and variance 3.27 < 8), whereas the mean and variance of the generalized *Q*‐statistics were 3.97 and 9.87 for $\sigma_{T}^{2}$ . The approximation was again best for sample sizes equal to 300 (dotted black line; mean of generalized *Q*‐statistics 3.90 and variance 7.41). Here, the coverage probability of the *Q*‐profile method (0.952) also approached the nominal coverage rate. These results indicate that a sample size of 30 was not sufficiently large to accurately approximate the *χ*^2^ distribution with ${\hat{\sigma }}^{2}$ when *k* = 5, $\pi_{i}^{C}=0.1$, and *τ* = 0. However, this approximation improved if $\sigma_{T}^{2}$ was used for computing the sampling variance or the sample size was equal to 300. Overcoverage of the methods for *k* = 5, $\pi_{i}^{C}=0.1$, *τ* = 0 and sample sizes equal to 30 can be explained by the distribution of the generalized *Q*‐statistic. Since the distribution of the generalized *Q*‐statistic is to the left of the *χ*^2^ distribution and its variance is smaller than that of the *χ*^2^ distribution, the CIs will too often include *τ* = 0.

For the condition with too low coverage probability (right panel; *k* = 160, $\pi_{i}^{C}=0.1$, *τ* = 0.5), the pdf of the generalized *Q*‐statistic based on estimator ${\hat{\sigma }}^{2}$ with a sample size of 30 per group (solid black line) deviated from the pdf of the *χ*^2^ statistic. The mean (117.10) and variance (124.12) of the generalized *Q*‐statistics were both substantially lower than those of a *χ*^2^ distribution with 159 degrees of freedom (mean 159 and variance 318).

The undercoverage was most severe for the condition with the largest value of *k*, because the distortion of the pdf of the generalized *Q*‐statistic accumulated if *k* increased. To illustrate this, we have computed the mean of the generalized *Q*‐statistics for $\pi_{i}^{C}=0.1$ and *τ* = 0.5 when *k* = 5, 10, 40, and 80, and divided it by the expected value of the *χ*^2^ distribution (which equals *k* − 1). These ratios were not varying much in *k* (0.757, 0.744, 0.738, 0.738), demonstrating that the bias (difference between mean of the generalized *Q*‐statistics and expected value of the *χ*^2^ distribution) increases approximately linearly in *k*. However, the SD of both the generalized *Q*‐statistics and *χ*^2^ distribution increases less strongly in *k*; more precisely, it increases approximately linearly in the square root of *k*, because the SD of the *χ*^2^ distribution is $\sqrt{2\left(k-1\right)}$. As a result, relative or standardized bias ([mean generalized *Q*‐statistic −*k* + 1]/SD) increases approximately linear with the square root of *k* as well, which explains why coverage probability started to deviate more from the nominal coverage rate if *k* increased.

Using ${\hat{\sigma }}_{T}^{2}$ instead of ${\hat{\sigma }}^{2}$ resulted in a pdf markedly closer to the pdf of the *χ*^2^ statistic. However, the generalized *Q*‐statistics computed with $\sigma_{T}^{2}$ (dashed gray line; mean 156.40 and variance 384.30) still deviated from those of the *χ*^2^ distribution. Again, increasing the primary studies' sample size to 300 yielded a pdf of the generalized *Q*‐statistic that was better approximated by the *χ*^2^ distribution (dotted black line; mean 153.90, variance 288.81). For this condition, the coverage probability of the *Q*‐profile method (0.945) was also close to the nominal coverage rate. These results suggest that for *k* = 160, $\pi_{i}^{C}=0.1$, and *τ* = 0.5, a sample size of 30 was too small to accurately approximate the *χ*^2^ distribution even if the true sampling variances were used ( $\sigma_{T}^{2}$).

Using the pdf of the generalized *Q*‐statistic, we can now explain the undercoverage of the *Q*‐profile method. Because the distribution of the *Q*‐statistic is to the left of the *χ*^2^ distribution, the lower and upper bounds of the CI around *τ* have to be obtained by decreasing *τ*^2^ in Equation 3 till the 2.5th and 97.5th percentiles of this *χ*^2^ distribution are reached. Consequently, the CIs of the *Q*‐profile method have too low lower and upper bounds, with *τ* often being larger than the upper bound. This was also apparent in the results of simulation study 1 in the condition *k* = 160, $\pi_{i}^{C}=0.1$, and *τ* = 0.5, because the lower bound was never lower than *τ* and the undercoverage was fully explained by the upper bound being often smaller than *τ*.

## 10 CONCLUSION AND DISCUSSION

Between‐study variance is often present in a meta‐analysis.^6^, ^9^, ^37^ The amount of between‐study variance can be estimated, but estimates are usually rather imprecise.^7^, ^8^ An estimate of the amount of between‐study variance can be surrounded by a CI to illustrate its imprecision. Two recommended methods^22^ to compute such a CI are the *Q*‐profile^18^ and GENQ method.^19^, ^20^ Both methods yield exact CIs under the assumptions of the random‐effects model (ie, unbiased observed effect size estimates, normal sampling distributions of the effect sizes, known sampling variances, and uncorrelated sampling errors and random effects). However, these assumptions are most likely violated in practice^14^, ^24^, ^25^ such that CIs of the *Q*‐profile and GENQ methods are approximations rather than exact CIs. The goal of the present paper is to study the performance of both methods under situations that are representative for research in practice where the assumptions underlying the random‐effects model are violated. This paper is the first that does not compare the performance of the *Q*‐profile and GENQ methods under the assumptions of the random‐effects model but uses conditions that are representative for meta‐analyses in practice where the assumptions of the random‐effects model are violated.

Results of two Monte‐Carlo simulation studies revealed that coverage probabilities of both methods can be substantially below the nominal coverage rate if model assumptions are violated. Coverage probabilities of both methods were especially too low if both the sample sizes of the primary studies and the probability of the outcome of interest were low in combination with a large number of studies in a meta‐analysis. This result is in line with Viechtbauer^18^ who also showed that the coverage probability of the *Q*‐profile method was too low if the number of studies was large in a meta‐analysis in combination with large between‐study heterogeneity.

Hence, we do not recommend to apply the *Q*‐profile and GENQ methods when the probability of the outcome of interest is low (ie, probability <0.1) in combination with 80 studies or more in a meta‐analysis and 40 studies or more when the primary studies sample size is small (ie, 30 observations per group or less). For these characteristics of a meta‐analysis, the coverage probabilities of the methods are no longer acceptable (ie, coverage <0.9). However, it is important to note that the effects of factors deteriorating the statistical properties of the methods (low probability of the outcome of interest, large number of studies in a meta‐analysis, and small number of observations per group) are synergetic, making it difficult to formulate general recommendations based on results of individual factors.

Coverage probabilities of the *Q*‐profile method and the GENQ method with variance weights were comparable in our simulation studies. If coverage of the *Q*‐profile and GENQ methods with variance weights was close to the nominal rate, coverage probability of the GENQ method with standard error weights deviated more from the nominal rate than the other two methods. However, the GENQ method with standard error weights yielded better coverage probabilities than the *Q*‐profile and GENQ methods with variance weights if the coverage probability of the *Q*‐profile and GENQ methods with variance weights was substantially too low. This was caused by the difference in weights, because more extreme observed log odds ratios (with larger sampling variances/standard errors) have a larger influence on the exact distribution of the *Q*‐statistic if standard error weights are used instead of variance weights. However, coverage probabilities of the methods substantially deviated from the nominal coverage rate if the probability of the outcome of interest was low.

Our second simulation study showed that the mean and variance of the sampling distribution of the generalized *Q*‐statistic may be too small in comparison to a *χ*^2^ distribution with *k* − 1 degrees of freedom if the probability of the outcome of interest was low and sample sizes were small. Consequently, the coverage probability of the *Q*‐profile method is too small in these conditions even if the true sampling variances instead of estimated sampling variances are used. This deviation from the nominal coverage rate is caused by low frequencies in some of the cells of the observed frequency tables. Specific methods have been developed that perform better in such cases with sparse data by analyzing dichotomous data by means of generalized linear mixed‐effects models. The sampling distributions in these methods are no longer assumed to be normal; instead, the exact likelihood based on binomial, Poisson, or hypergeometric distributions is used.^38^ This approach is especially beneficial in case of a low probability of the outcome of interest, because no corrections (eg, adding 0.5 to each cell) are required to deal with zero cells. However, future research is still needed to determine under which conditions the generalized linear mixed‐effects models have better statistical properties for constructing CIs for *τ*^2^ than the *Q*‐profile and GENQ methods.

A CI around the estimate of the between‐study variance can also be used for computing a CI around the *I* ^2^ statistic (ie, proportion of the total variance in a meta‐analysis caused by the between‐study variance).^39^ Hence, the results presented in this paper also apply to CIs around the *I* ^2^ statistic if constructed with the *Q*‐profile or GENQ method. An advantage of quantifying between‐study heterogeneity with the *I* ^2^ statistic is that it enables comparisons across meta‐analyses.^3^, ^39^ CIs around the estimate of between‐study variance and the *I* ^2^ statistic can also be used for testing the null hypothesis of homogeneous effect sizes in a meta‐analysis. Software for applying the *Q*‐profile and GENQ methods for estimating a CI around the estimate of the between‐study variance and the *I* ^2^ statistic is readily available in the R package *metafor*.^36^

The commonly used *Q*‐test^2^ for testing the null hypothesis of homogeneous effect sizes in a meta‐analysis is also based on the assumptions of the random‐effects model. The *Q*‐statistic follows a *χ*^2^ distribution if the assumptions underlying the random‐effects model hold. Hence, inferences drawn by using the *Q*‐test will also be affected by violations of these assumptions as is the case for the *Q*‐profile and GENQ methods, but the assumptions become more acceptable if the primary studies' sample size increase. Similar to our results with respect to the generalized *Q*‐statistic, Kulinskaya and Dollinger^40^ showed that the mean and variance of the distribution of the *Q*‐statistic are too low when log odds ratios are used as effect size measure and the sample size is not sufficiently large. They propose to approximate the distribution of the *Q*‐statistic by means of a gamma distribution and developed a new test for homogeneity based on this approximation. Future research may study whether the statistical properties of the *Q*‐profile method improve if the distribution of the generalized *Q*‐statistic is approximated by a gamma distribution instead of a *χ*^2^ distribution.

Future research may also examine to what extent incorporating an estimate of the between‐study variance in the weights of the GENQ method affects its CIs if the assumptions underlying the random‐effects model do not hold. Using variance weights where an estimate of the between‐study variance is also included corresponds to the standard weights that are commonly used in the random‐effects model. However, the GENQ method is no longer exact if such an estimate is incorporated. Jackson^20^ already studied the statistical properties of the GENQ method when incorporating an estimate of the between‐study variance in the weights, but only when the assumptions of the random‐effects model hold; he concluded that the coverage probability only slightly deviated from the nominal coverage rate under these conditions.

One limitation of our paper is that we only focus on one particular effect size measure (odds ratio) in our Monte‐Carlo simulation studies. In order to examine the dependency of the results on the type of effect size measure used, we conducted two small‐scale Monte‐Carlo simulation studies, one with risk difference and one with standardized mean difference (ie, Hedges' *g*) as effect size measure (for details and results see https://osf.io/643hv/ and https://osf.io/4qd7b/). For risk difference as effect size measure, coverage probabilities of all methods were substantially below the nominal coverage rate if the true effect size was heterogeneous. Statistical properties for risk difference were worse than for the odds ratio, where, for instance, coverage was close to the nominal coverage rate if the probability on the outcome of interest was 0.5. Coverage probability of the methods with standardized mean difference as effect size measure was close to the nominal coverage rate in all conditions. This was in line with our expectation, because the sampling distribution of a standardized mean difference more closely follows a normal distribution than that of the effect size measures odds ratio and risk difference. Hence, using standardized mean difference as effect size measure is more in line with the assumptions of random‐effects meta‐analysis. Since our results clearly show that the statistical properties of the *Q*‐profile and GENQ methods are effect size measure dependent, future research may therefore especially examine the statistical properties of both methods for effect size measures whose sampling distribution deviates from a normal distribution and formulates conditions under which these methods perform well.

To conclude, between‐study heterogeneity is common in meta‐analyses,^41^, ^42^ and assessing heterogeneity is a crucial issue.^43^ We recommend in line with others^6^, ^10^, ^11^, ^12^ to include a CI around the estimate of between‐study variance computed with the *Q*‐profile or GENQ method in every meta‐analysis. This illustrates imprecision in the estimate of the between‐study variance and facilitates interpretation of the meta‐analysis. Previous research has shown that the *Q*‐profile and GENQ methods have the best statistical properties, but the methods' coverage probabilities deviate from the nominal coverage rate if the probability of the outcome of interest is small. Hence, methods specifically developed for those situations should be considered to be used instead of the *Q*‐profile or GENQ method.

## Supporting information

Table S1. For Monte‐Carlo simulation study 1, the proportion of simulation runs where a meta‐analysis contained at least one primary study with a zero cell (Zero), coverage rate of the methods based on simulation runs where a meta‐analysis contained at least one primary study with a zero cell (Coverage zeros), and coverage rate of the methods based on only the simulation runs where the primary studies in a meta‐analysis did not contain a zero cell (Coverage without zeros). GENQ (var.) is the GENQ method with variance weights ( $a_{i}=1/{\hat{\sigma }}_{i}^{2}$) and GENQ (SE) is the GENQ method with standard error weights (SE; $a_{i}=1/{\hat{\sigma }}_{i}$). Results are shown for the condition *μ* = 0 and $\pi_{i}^{C}=0.1$.
Table S2. For Monte‐Carlo simulation study 2, the proportion of simulation runs where a meta‐analysis contained at least one primary study with a zero cell (Zero), coverage rate of the methods using ${\hat{\sigma }}^{2}$ as estimator for the sampling variance based on simulation runs where a meta‐analysis contained at least one primary study with a zero cell (Coverage zeros), and only for the simulation runs where the primary studies in a meta‐analysis did not contain a zero cell (Coverage without zeros). GENQ (var.) is the GENQ method with variance weights ( $a_{i}=1/{\hat{\sigma }}_{i}^{2}$) and GENQ (SE) is the GENQ method with standard error weights (SE; $a_{i}=1/{\hat{\sigma }}_{i}$). Results are shown for the condition *μ* = 0 and $\pi_{i}^{C}=0.1$.
Table S3. For Monte‐Carlo simulation study 2, the proportion of simulation runs where a meta‐analysis contained at least one primary study with two zero cells (Zeros), coverage rate of the methods using ${\hat{\sigma }}^{2}$ as estimator for the sampling variance based on simulation runs where a meta‐analysis contained at least one primary study with two zero cells (Coverage zeros), and only for the simulation runs where the primary studies in a meta‐analysis did not contain two zero cells (Coverage without zeros). GENQ (var.) is the GENQ method with variance weights ( $a_{i}=1/{\hat{\sigma }}_{i}^{2}$) and GENQ (SE) is the GENQ method with standard error weights (SE; $a_{i}=1/{\hat{\sigma }}_{i}$). Results are shown for the condition *μ* = 0 and $\pi_{i}^{C}=0.1$.
