# Supplementary material for: Statistical properties of methods based on the Q‐statistic for constructing a confidence interval for the between‐study variance in meta‐analysis
Source: Res Synth Methods. 2019 Jan 28;10(2):225–39. doi: 10.1002/jrsm.1336 (PMC6590162; doi:10.1002/jrsm.1336)
Supplement: Supplementary file 1 — Table S1. For Monte‐Carlo simulation study 1, the proportion of simulation runs where a meta‐analysis contained at least one primary study with a zero cell (Zero), coverage rate of the methods based on simulation runs where a meta‐analysis contained at least one primary study with a zero cell (Coverage zeros), and coverage rate of the methods based on only the simulation runs where the primary studies in a meta‐analysis did not contain a zero cell (Coverage without zeros). GENQ (var.) is the GENQ method with variance weights ( ai=1/σ^i2) and GENQ (SE) is the GENQ method with standard error weights (SE; ai=1/σ^i). Results are shown for the condition μ = 0 and πiC=0.1. Table S2. For Monte‐Carlo simulation study 2, the proportion of simulation runs where a meta‐analysis contained at least one primary study with a zero cell (Zero), coverage rate of the methods using σ^2 as estimator for the sampling variance based on simulation runs where a meta‐analysis contained at least one primary study with a zero cell (Coverage zeros), and only for the simulation runs where the primary studies in a meta‐analysis did not contain a zero cell (Coverage without zeros). GENQ (var.) is the GENQ method with variance weights ( ai=1/σ^i2) and GENQ (SE) is the GENQ method with standard error weights (SE; ai=1/σ^i). Results are shown for the condition μ = 0 and πiC=0.1. Table S3. For Monte‐Carlo simulation study 2, the proportion of simulation runs where a meta‐analysis contained at least one primary study with two zero cells (Zeros), coverage rate of the methods using σ^2 as estimator for the sampling variance based on simulation runs where a meta‐analysis contained at least one primary study with two zero cells (Coverage zeros), and only for the simulation runs where the primary studies in a meta‐analysis did not contain two zero cells (Coverage without zeros). GENQ (var.) is the GENQ method with variance weights ( ai=1/σ^i2) and GENQ (SE) is the GENQ method with standard error weights (SE [file JRSM-10-225-s001.docx]

**Supporting information**

Statistical properties of methods based on the *Q-*statistic for constructing a confidence interval for the between-study variance in meta-analysis

Robbie C. M. van Aert, Marcel A. L. M. van Assen, and Wolfgang Viechtbauer

*Table S1.* For Monte-Carlo simulation study 1, the proportion of simulation runs where a meta-analysis contained at least one primary study with a zero cell (Zero), coverage rate of the methods based on simulation runs where a meta-analysis contained at least one primary study with a zero cell (Coverage zeros), and coverage rate of the methods based on only the simulation runs where the primary studies in a meta-analysis did not contain a zero cell (Coverage without zeros). GENQ (var.) is the GENQ method with variance weights ($a_{i}=1/\hat{\sigma}_{i}^{2}$) and GENQ (SE) is the GENQ method with standard error weights (SE; $a_{i}=1/\hat{\sigma}_{i}$). Results are shown for the condition $\mu=0$ and $\pi_{i}^{C}=0.1$.

|  |  |  | Coverage zeros | | | Coverage nonzero | | |
| --- | --- | --- | --- | --- | --- | --- | --- | --- |
|  | $\tau$ | Zero | *Q-*profile | GENQ (var.) | GENQ (SE) | *Q-*profile | GENQ (var.) | GENQ (SE) |
| *k* = 5 | 0 | 0.092 | 0.985 | 0.985 | 0.985 | 0.966 | 0.966 | 0.968 |
|  | 0.1 | 0.089 | 0.984 | 0.983 | 0.992 | 0.962 | 0.962 | 0.965 |
|  | 0.2 | 0.101 | 0.985 | 0.985 | 0.985 | 0.959 | 0.958 | 0.963 |
|  | 0.3 | 0.105 | 0.98 | 0.972 | 0.988 | 0.96 | 0.957 | 0.962 |
|  | 0.4 | 0.116 | 0.979 | 0.98 | 0.982 | 0.962 | 0.959 | 0.964 |
|  | 0.5 | 0.134 | 0.982 | 0.975 | 0.982 | 0.961 | 0.959 | 0.961 |
| *k* = 10 | 0 | 0.171 | 0.982 | 0.982 | 0.994 | 0.963 | 0.963 | 0.964 |
|  | 0.1 | 0.181 | 0.985 | 0.983 | 0.994 | 0.958 | 0.957 | 0.963 |
|  | 0.2 | 0.19 | 0.98 | 0.975 | 0.987 | 0.963 | 0.961 | 0.965 |
|  | 0.3 | 0.202 | 0.979 | 0.972 | 0.987 | 0.958 | 0.954 | 0.959 |
|  | 0.4 | 0.219 | 0.981 | 0.973 | 0.984 | 0.955 | 0.954 | 0.957 |
|  | 0.5 | 0.244 | 0.985 | 0.971 | 0.985 | 0.953 | 0.95 | 0.955 |
| *k* = 40 | 0 | 0.541 | 0.953 | 0.953 | 0.979 | 0.929 | 0.929 | 0.933 |
|  | 0.1 | 0.541 | 0.96 | 0.959 | 0.981 | 0.923 | 0.922 | 0.933 |
|  | 0.2 | 0.57 | 0.954 | 0.95 | 0.976 | 0.919 | 0.92 | 0.929 |
|  | 0.3 | 0.6 | 0.952 | 0.945 | 0.97 | 0.927 | 0.925 | 0.935 |
|  | 0.4 | 0.642 | 0.951 | 0.939 | 0.964 | 0.909 | 0.914 | 0.911 |
|  | 0.5 | 0.685 | 0.948 | 0.932 | 0.958 | 0.907 | 0.913 | 0.91 |
|  |  |  |  |  |  |  |  |  |
| *Table S1.* Continued | | |  |  |  |  |  |  |
|  |  |  | Coverage zeros | | | Coverage nonzero | | |
|  | $\tau$ | Zero | *Q-*profile | GENQ (var.) | GENQ (SE) | *Q-*profile | GENQ (var.) | GENQ (SE) |
| *k* = 80 | 0 | 0.788 | 0.922 | 0.922 | 0.962 | 0.884 | 0.884 | 0.887 |
|  | 0.1 | 0.791 | 0.918 | 0.916 | 0.954 | 0.865 | 0.863 | 0.877 |
|  | 0.2 | 0.815 | 0.912 | 0.911 | 0.949 | 0.863 | 0.868 | 0.868 |
|  | 0.3 | 0.834 | 0.913 | 0.903 | 0.945 | 0.877 | 0.885 | 0.873 |
|  | 0.4 | 0.865 | 0.909 | 0.897 | 0.935 | 0.879 | 0.875 | 0.876 |
|  | 0.5 | 0.906 | 0.904 | 0.882 | 0.926 | 0.851 | 0.871 | 0.841 |
| *k* = 160 | 0 | 0.956 | 0.841 | 0.841 | 0.913 | 0.796 | 0.796 | 0.754 |
|  | 0.1 | 0.958 | 0.844 | 0.844 | 0.909 | 0.796 | 0.796 | 0.766 |
|  | 0.2 | 0.963 | 0.842 | 0.843 | 0.904 | 0.761 | 0.762 | 0.742 |
|  | 0.3 | 0.978 | 0.828 | 0.819 | 0.888 | 0.737 | 0.728 | 0.752 |
|  | 0.4 | 0.984 | 0.828 | 0.816 | 0.877 | 0.703 | 0.691 | 0.689 |
|  | 0.5 | 0.989 | 0.809 | 0.783 | 0.848 | 0.718 | 0.692 | 0.757 |

*Table S2.* For Monte-Carlo simulation study 2, the proportion of simulation runs where a meta-analysis contained at least one primary study with a zero cell (Zero), coverage rate of the methods using $\hat{\sigma}^{2}$ as estimator for the sampling variance based on simulation runs where a meta-analysis contained at least one primary study with a zero cell (Coverage zeros), and only for the simulation runs where the primary studies in a meta-analysis did not contain a zero cell (Coverage without zeros). GENQ (var.) is the GENQ method with variance weights ($a_{i}=1/\hat{\sigma}_{i}^{2}$) and GENQ (SE) is the GENQ method with standard error weights (SE; $a_{i}=1/\hat{\sigma}_{i}$). Results are shown for the condition $\mu=0$ and $\pi_{i}^{C}=0.1$.

|  |  |  | Coverage zeros | | | Coverage nonzero | | |
| --- | --- | --- | --- | --- | --- | --- | --- | --- |
|  | $\tau$ | Zero | *Q-*profile | GENQ (var.) | GENQ (SE) | *Q-*profile | GENQ (var.) | GENQ (SE) |
| *k* = 5 | 0 | 0.358 | 0.991 | 0.991 | 0.996 | 0.963 | 0.963 | 0.965 |
|  | 0.1 | 0.332 | 0.991 | 0.991 | 0.994 | 0.964 | 0.964 | 0.966 |
|  | 0.2 | 0.377 | 0.992 | 0.992 | 0.994 | 0.958 | 0.958 | 0.96 |
|  | 0.3 | 0.386 | 0.996 | 0.996 | 0.996 | 0.962 | 0.96 | 0.96 |
|  | 0.4 | 0.413 | 0.993 | 0.993 | 0.993 | 0.952 | 0.952 | 0.95 |
|  | 0.5 | 0.454 | 0.991 | 0.99 | 0.99 | 0.954 | 0.955 | 0.955 |
| *k* = 40 | 0 | 0.967 | 0.853 | 0.853 | 0.931 | 0.611 | 0.611 | 0.658 |
|  | 0.1 | 0.972 | 0.861 | 0.86 | 0.934 | 0.647 | 0.61 | 0.684 |
|  | 0.2 | 0.978 | 0.847 | 0.84 | 0.915 | 0.62 | 0.658 | 0.688 |
|  | 0.3 | 0.981 | 0.847 | 0.83 | 0.92 | 0.531 | 0.567 | 0.604 |
|  | 0.4 | 0.984 | 0.837 | 0.807 | 0.905 | 0.524 | 0.494 | 0.53 |
|  | 0.5 | 0.99 | 0.819 | 0.765 | 0.88 | 0.419 | 0.465 | 0.48 |
| *k* = 80 | 0 | 0.999 | 0.633 | 0.633 | 0.84 | 0.633 | 0.633 | 0.84 |
|  | 0.1 | 1 | 0.651 | 0.646 | 0.855 | - | - | - |
|  | 0.2 | 1 | 0.634 | 0.613 | 0.843 | - | - | - |
|  | 0.3 | 1 | 0.62 | 0.576 | 0.822 | - | - | - |
|  | 0.4 | 1 | 0.582 | 0.504 | 0.771 | - | - | - |
|  | 0.5 | 1 | 0.582 | 0.467 | 0.74 | - | - | - |
|  |  |  |  |  |  |  |  |  |
| *Table S2.* Continued | | |  |  |  |  |  |  |
|  |  |  | Coverage zeros | | | Coverage nonzero | | |
|  | $\tau$ | Zero | *Q-*profile | GENQ (var.) | GENQ (SE) | *Q-*profile | GENQ (var.) | GENQ (SE) |
| *k* = 160 | 0 | 1 | 0.265 | 0.265 | 0.649 | - | - | - |
|  | 0.1 | 1 | 0.262 | 0.257 | 0.657 | - | - | - |
|  | 0.2 | 1 | 0.252 | 0.228 | 0.629 | - | - | - |
|  | 0.3 | 1 | 0.229 | 0.178 | 0.587 | - | - | - |
|  | 0.4 | 1 | 0.231 | 0.157 | 0.526 | - | - | - |
|  | 0.5 | 1 | 0.22 | 0.115 | 0.471 | - | - | - |

*Note. -* indicates that there were no simulation runs without zeros for this condition

*Table S3.* For Monte-Carlo simulation study 2, the proportion of simulation runs where a meta-analysis contained at least one primary study with two zero cells (Zeros), coverage rate of the methods using $\hat{\sigma}^{2}$ as estimator for the sampling variance based on simulation runs where a meta-analysis contained at least one primary study with two zero cells (Coverage zeros), and only for the simulation runs where the primary studies in a meta-analysis did not contain two zero cells (Coverage without zeros). GENQ (var.) is the GENQ method with variance weights ($a_{i}=1/\hat{\sigma}_{i}^{2}$) and GENQ (SE) is the GENQ method with standard error weights (SE; $a_{i}=1/\hat{\sigma}_{i}$). Results are shown for the condition $\mu=0$ and $\pi_{i}^{C}=0.1$.

|  |  |  | Coverage zeros | | | Coverage nonzero | | |
| --- | --- | --- | --- | --- | --- | --- | --- | --- |
|  | $\tau$ | Zeros | *Q-*profile | GENQ (var.) | GENQ (SE) | *Q-*profile | GENQ (var.) | GENQ (SE) |
| *k* = 5 | 0 | 0.008 | 0.84 | 0.84 | 0.88 | 0.974 | 0.974 | 0.977 |
|  | 0.1 | 0.011 | 0.912 | 0.912 | 0.912 | 0.974 | 0.974 | 0.976 |
|  | 0.2 | 0.014 | 0.905 | 0.905 | 0.905 | 0.972 | 0.972 | 0.974 |
|  | 0.3 | 0.008 | 1 | 1 | 1 | 0.975 | 0.974 | 0.974 |
|  | 0.4 | 0.012 | 0.889 | 0.889 | 0.861 | 0.97 | 0.97 | 0.969 |
|  | 0.5 | 0.014 | 0.881 | 0.881 | 0.881 | 0.972 | 0.972 | 0.972 |
| *k* = 40 | 0 | 0.072 | 0.823 | 0.823 | 0.879 | 0.847 | 0.847 | 0.925 |
|  | 0.1 | 0.074 | 0.798 | 0.798 | 0.897 | 0.86 | 0.857 | 0.929 |
|  | 0.2 | 0.078 | 0.811 | 0.803 | 0.85 | 0.845 | 0.839 | 0.915 |
|  | 0.3 | 0.081 | 0.789 | 0.764 | 0.847 | 0.846 | 0.83 | 0.92 |
|  | 0.4 | 0.102 | 0.785 | 0.759 | 0.84 | 0.837 | 0.807 | 0.906 |
|  | 0.5 | 0.111 | 0.796 | 0.731 | 0.853 | 0.817 | 0.766 | 0.879 |
| *k* = 80 | 0 | 0.148 | 0.616 | 0.616 | 0.822 | 0.636 | 0.636 | 0.843 |
|  | 0.1 | 0.128 | 0.592 | 0.592 | 0.792 | 0.66 | 0.654 | 0.864 |
|  | 0.2 | 0.155 | 0.592 | 0.582 | 0.781 | 0.642 | 0.618 | 0.854 |
|  | 0.3 | 0.167 | 0.601 | 0.573 | 0.782 | 0.624 | 0.577 | 0.83 |
|  | 0.4 | 0.182 | 0.553 | 0.491 | 0.729 | 0.588 | 0.507 | 0.78 |
|  | 0.5 | 0.218 | 0.541 | 0.432 | 0.674 | 0.593 | 0.477 | 0.758 |
|  |  |  |  |  |  |  |  |  |
| *Table S3.* Continued | | |  |  |  |  |  |  |
|  |  |  | Coverage zeros | | | Coverage nonzero | | |
|  | $\tau$ | Zeros | *Q-*profile | GENQ (var.) | GENQ (SE) | *Q-*profile | GENQ (var.) | GENQ (SE) |
| *k* = 160 | 0 | 0.251 | 0.265 | 0.265 | 0.61 | 0.265 | 0.265 | 0.662 |
|  | 0.1 | 0.246 | 0.251 | 0.247 | 0.629 | 0.266 | 0.26 | 0.666 |
|  | 0.2 | 0.279 | 0.247 | 0.232 | 0.571 | 0.254 | 0.226 | 0.651 |
|  | 0.3 | 0.31 | 0.214 | 0.172 | 0.534 | 0.236 | 0.181 | 0.611 |
|  | 0.4 | 0.337 | 0.207 | 0.144 | 0.492 | 0.243 | 0.164 | 0.543 |
|  | 0.5 | 0.37 | 0.221 | 0.122 | 0.434 | 0.219 | 0.111 | 0.493 |
